# Capillary Glycated Hemoglobin A1c Percentiles and the Risk Factors Associated with Abnormal HbA1c among Chinese Children Aged 3–12 Years

**DOI:** 10.1155/2024/8333590

**Published:** 2024-07-29

**Authors:** Longbing Ren, Jing Yang, Lezhou Wu, Yan Gao, Zhitong Zhou, Pin Li, Zhiping Shen, Juanli Wu, Jue Li, Lijuan Zhang

**Affiliations:** ^1^ Clinical Center for Intelligent Rehabilitation Research Shanghai YangZhi Rehabilitation Hospital (Shanghai Sunshine Rehabilitation Center) Tongji University School of Medicine Tongji University, Shanghai 201613, China; ^2^ Community Health Service Center of Anting Town Affiliated with Medical College of Tongji University, Shanghai 201805, China; ^3^ Department of Biomedical and Health Informatics Children's Hospital of Philadelphia, Philadelphia 19147, PA, USA; ^4^ Children's Hospital of Fudan University, 399 Wan Yuan Road, Shanghai 201102, China; ^5^ Daqiao Community Health Service Center Tongji University School of Medicine Shanghai, Shanghai 200090, China

## Abstract

**Objective:**

Capillary glycated hemoglobin (HbA1c) may enhance screening for childhood prediabetes and diabetes, but variations in this parameter remain unclear. We aimed to develop percentiles of HbA1c and explore the influence of various variables on HbA1c level among Chinese children. *Study Design and Methods*. The data were derived from the Shanghai Children's Health and Nutrition Community-based Epidemiologic Survey (CHANCE). A total of 4,615 children aged 3–12 years were included. The capillary HbA1c level was measured using point-of-care (POC) testing analyzers. Abnormal HbA1c level was identified as HbA1c (%) value equal to or above the 95th percentile of the nomograms.

**Results:**

The mean HbA1c value was 5.30% (SD = 0.50%). The age-specific 95th percentile thresholds of HbA1c (%) ranged from 5.9 to 6.2 among all children. In the whole participants, body mass index (BMI), total cholesterol (TC), outdoor activity frequency, and daily sleep duration were positively associated with high HbA1c. Among preschool-aged children, TC and sleep duration ≥10 hr per day were associated with increased risk of being in the higher HbA1c (both *P* < 0.05). Among the school-aged group, positive associations with HbA1c levels were identified for TC, living with grandparents, frequency of outdoor activity, and sleep duration (all *P* < 0.05).

**Conclusions:**

The present study established capillary HbA1c percentiles based on a large sample of Chinese children among aged 3–12 years. Daily sleep duration and frequency of outdoor activity, BMI, and TC were found to be associated with high HbA1c. Actions of successful public strategies that focus on promoting a healthy lifestyle, including regular physical exercise to reduce weight among children, are needed. *Key Points*. We used POC testing for capillary HbA1c with a finger-stick sample which may offer an opportunity to enhance screening and early diagnosis for childhood and adolescent diabetes, which was suggested as an essential premise to determine the subject's glycemic status by the American Diabetes Association (ADA). Capillary HbA1c levels fluctuate during childhood, while there has been no population-based study on HbA1c reference values in Chinese youths.

## 1. Introduction

Diabetes in childhood and adolescence poses a significant clinical and public health challenge worldwide [1, 2, 3, 4, 5, 6, 7, 8]. According to the 2020 National Diabetes Statistics Report by the Centers for Disease Control and Prevention [2], 25 per 10,000 American youths under 20 years old were diagnosed with diabetes. An estimated annual increase of 6.4% in diabetes incidence was detected among American children and adolescents [3, 4]. Similar increasing incidence trends were also observed in European countries and China [5, 6, 7, 8]. To address the global diabetes epidemic among children and adolescents, some key efforts have been made including active screening, timely diagnosis, long-term monitoring, and management of diabetes at an early age, which may help detect high-risk youngsters and then prevent or slow the onset of clinical symptoms and complications.

Diabetes can be assessed using fasting plasma glucose (FPG), a 2-hr plasma glucose (2-hr PG) during a 75-g oral glucose tolerance test (OGTT), or glycated hemoglobin (HbA1c). Compared with FPG and OGTT, HbA1c is advantageous because it reflects cumulative glycemic status over months, has better preanalytical stability, and causes fewer acute perturbations related to stress and illness [9, 10]. However, the current HbA1c diagnostic criteria for prediabetes (HbA1c : 5.7%–6.4%) and diabetes (HbA1c ≥ 6.5%) are only recommended for adults, and the evidence-based reference ranges for HbA1c in children and adolescents remain unclear [9, 11, 12]. This gap in knowledge causes uncertainty, possibly exposing many youths to an increased risk of delayed diagnosis and nonstandardized care. Point-of-care (POC) testing for capillary HbA1c with a finger-stick sample may offer an opportunity to enhance screening and early diagnosis for childhood and adolescent diabetes, and this method was suggested to be essential for diagnosing diabetes by the American Diabetes Association (ADA) [9].

Our scientific colleagues rigorously validated a POC HbA1c analyzer [13, 14], and in the present study, we conducted a prospective epidemiology study aimed at evaluating capillary HbA1c percentiles based on a large sample of healthy children aged 3–12 years and explored the influence of various variables on HbA1c values among these children.

## 2. Methods

### 2.1. Study Design and Population

**Figure 1:**
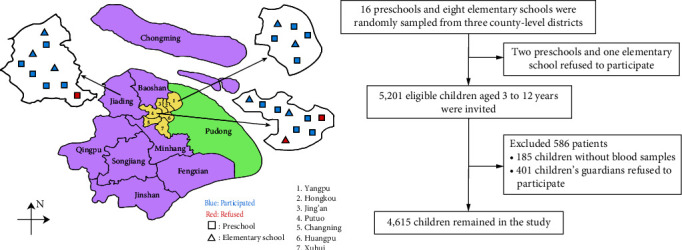
The multistage geographic cluster sampling and the recruitment of study participants, Shanghai, China, 2018–2019. Shanghai is divided into 16 districts with yellow, purple, and green indicating downtown districts, suburban districts, and semi-urban and semi-suburban district, respectively.

The Children's Health and Nutrition Community-based Epidemiologic Survey (CHANCE) of Tongji University is a prospective longitudinal population health study jointly initiated by the Tongji University School of Medicine and five primary healthcare centers in Shanghai, China. The first stage of the Shanghai CHANCE survey was conducted from January 2018 to December 2019. A multistage cluster sampling approach was used to recruit target children aged 3–12 years who attended local preschools or elementary schools. Of the 15 county-level districts in Shanghai (excluded Pudong New Area), one suburban and two urban districts were initially selected, within which a total of 24 schools were randomly sampled with at a ratio of two preschools per elementary school. Three schools refused to participate because of concerns about insufficient organizational capacity resulting in a school-level agreement rate of 87.5% (21/24). Of the 5,201 eligible children who were invited, 4,615 ultimately participated in the survey with valid blood samples resulting in an individual-level response rate of 88.7% (4,615/5,201). The details of the sampling process are shown in Figure 1 and *Supplementary table 1*. The study was approved by the Institutional Review Board of Tongji University, and informed consent was obtained from all participants and their parents.

This cross-sectional study was a primary analysis of the survey data. The study's initial inclusion and exclusion criteria, definitions of high HbA1c, and analysis plan were established before the data were accessed. The inclusion criteria were as follows: children who were aged between 3 and 12 years and whose HbA1c levels were measured during the study period. The exclusion criteria included: (1) children with known diabetes or other metabolic diseases; (2) children with chromosomal abnormalities such as Turner syndrome; (3) children with serious health problems such as congenital heart disease, cancer, and renal insufficiency; (4) children with mental health disorders; (5) children who received hormone treatment in the past year that might affect A1C levels, such as growth hormone and sex hormone; and (6) children with conditions associated with abnormal hemoglobin levels including sickle cell disease, anemia, recent blood loss or transfusion, glucose-6-phosphate dehydrogenase deficiency, and erythropoietin therapy.

### 2.2. Primary Outcome

Capillary HbA1c was measured using an A1C EZ 2.0 POC analyzer (BioHermes Ltd., Wuxi, Jiangsu, China). The POC test device was certified by the National Glycohemoglobin Standardization Program (NGSP) [15] and standardized to the Diabetes Control and Complications Trial (DCCT) reference assay. Using boric acid affinity chromatography, the POC results for HbA1c were compared with those multiple high-performance liquid chromatography (HPLC) devices with high accuracy and precision that have been extensively validated: a concordance correlation coefficient of 0.978 and a regression coefficient of 0.994 were reported compared with the Tosoh G8 HPLC analyzer [13]; mean relative differences of 2.5%, 0.6%, and 0.4% were reported when compared with three HPLC devices, Bio-Rad Variant II Turbo, Tosoh G8, and Premier Hb9210, respectively [14]. The evaluation of precision demonstrated the reproducibility of the total coefficient of variation (CVT), which ranged from 2.3%–3.7% to 1.9%–2.7% for low and high HbA1c samples, respectively [14]. Fingertip blood samples were collected, and fasting was not required prior to the test. In the initial analysis plan, high HbA1c was defined as an HbA1c (%) value equal to or greater than the 95th percentile of the age- and sex-specific nomograms derived from the study population.

**Figure 2:**
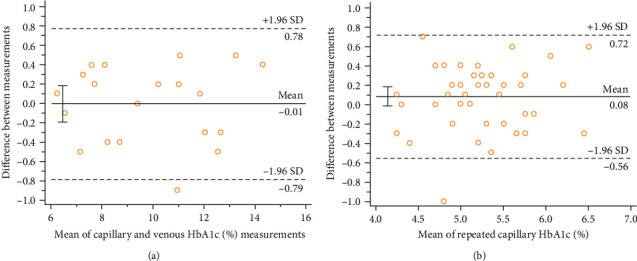
The Bland–Altman plot assessment for the reliability of POC testing for HbA1c (%). (a) Capillary versus venous blood specimens among 20 diabetic patients; (b) test–retest reliability of capillary HbA1c in a random subsample of 46 study participants. The differences of the two tests were plotted against the averages of two tests. Horizontal dotted lines were drawn at the limits of agreement, which were defined as the mean difference ±1.96 times the standard deviation of the differences.

We additionally performed two reliability assessments for the POC HbA1c test. First, (a) a pilot assessment was conducted to compare the POC test results using capillary versus venous blood specimens among 20 diabetic patients (11 boys and nine girls; average age 8.3 years with an age range 3–12 years) hospitalized at the Children's Hospital of Fudan University, an urban, tertiary care pediatric hospital in Shanghai, China. (b) The POC test–retest reliability of capillary HbA1c was assessed in a random subsample of 46 study participants (approximately 1% of the study population; 23 boys and 23 girls; average age 7.4 years with an age range of 3–12 years); the retest was completed 5 min after the initial one. The concordance correlation coefficients were 0.907 and 0.993, respectively (both *P*  < 0.001). The reliability assessments are also visualized in Figure 2 using the Bland–Altman plot: 95.0% (19/20) and 97.8% (45/46) of the pairs of repeated measures fell within 1.96 standard deviations of the mean difference of all pairs.

### 2.3. Sociodemographic and Health Features

Sociodemographic and health features were collected using a face-to-face interviewer-administered questionnaire. The questionnaire elements that were relevant to the study included sex, age, ethnicity, residential area, household income, and parental diabetes history. During the physical examination, weight, height, waist circumference, and hip circumference were measured. Body mass index (BMI) was calculated as weight in kilograms divided by height in meters squared (kg/m^2^). Capillary blood lipids, including total cholesterol (TC), triglyceride (TG), serum high-density lipoprotein (HDL) cholesterol, and serum low-density lipoprotein (LDL) cholesterol, were measured.

#### 2.3.1. Statistical Analysis

Descriptive statistics were performed to reveal the distribution of baseline characteristics, including sociodemographic features, physical examination features, laboratory indicators, family medical history, and behavioral features. The categorical and numeric variables are presented as counts with percentages and means with standard deviations or medians with interquartile ranges, respectively.

The age- and sex-specific percentile curves of HbA1c were fitted using the general additive model for location scale and shape (GAMLSS) approach. GAMLSS is a modern distribution-based semiparametric regression approach, with the flexibility to model linear, nonlinear, or smooth functions conditional on distribution parameters such as location (e.g., mean), scale (e.g., variance), and shape (e.g., skewness and kurtosis) parameters [11, 16, 17]. In this study, the GAMLSS model was stratified by sex with a fitted Box–Cox T- (BCT) distribution specifying two of four parameters (*μ*, *σ*, *ν*, and *τ*): the location, *μ*, was parameterized as a cubic spline function of age; the scale, *σ*, was parameterized as a log-linear function of age; and the shape parameters, *ν* and *τ*, remained constant. Given parameters, age- and sex-specific percentiles of HbA1c were computed.

To explore the influence of various variables on HbA1c values, the variables statistically significant in the univariate analysis were further included into multivariate analysis. Logistic regression analyses were performed, and odds ratio (OR) with 95% confidence interval (CI) is reported in the forest plot.

We performed subgroup analysis to explore factors associated with HbA1c percentiles varied by age. Sensitivity analyses were also conducted to test the robustness of the results, dividing children into different subgroups according to age.

All analyses were performed using R 3.4.2 (R Foundation for Statistical Computing, Austria). The R package gamlss was used for fitting reference percentile values and curves. A two-sided *P* value less than 0.05 was used as the criterion for statistical significance.

## 3. Results

### 3.1. Descriptive Statistics

The final analytic sample consisted of 4,615 participants. Of these, 1,413 (30.6%) were aged 3–5 years, and 3,202 (69.4%) were aged 6–12 years. Characteristics of the participants were shown in Table 1. In the present sample of children, 50.3% were boys, 49.7% were girls, and the median age was 7.3 (IQR = 4.7) years (range 2.9–12.7 years). Approximately 98.3% of these participants were Han Chinese, 74% of the total children resided in urban areas of Shanghai, 77.1% had a normal weight, and the median BMI was 16.3 (IQR = 3.4) kg/m^2^. The mean HbA1c value was 5.3% ± 0.5%. The most prevalent parental-reported disease was obesity (15.6%). Approximately 2.8% of these children had a parental history of diabetes. Information of participants' behaviors was also listed in Table 1. Most participants had a high-sugar diet habit (43.6%), a duration of 8–10 hr of daily sleep (66.0%), and slept at 8–10 p.m. (84.4%). More details of the anthropometric and HbA1c distributions by sex and age group are presented in *Supplementary table 2*.

### 3.2. Differences in HbA1c Percentiles: Gender and Age

**Figure 3:**
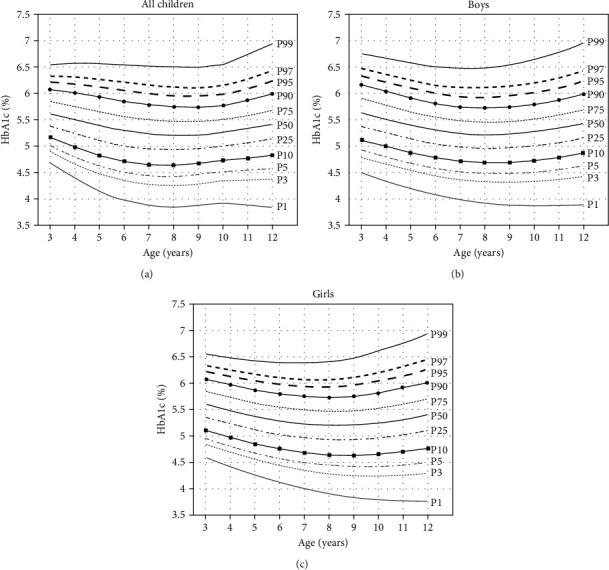
Reference percentile curves of HbA1c (%) derived from study participants aged 3–12 years, Shanghai, China, 2018–2019. (a) is age-specific reference percentile curves of HbA1c (%) derived from all study participants. (b and c) are sex- and age-specific reference percentile curves of HbA1c (%) derived from all study participants.

The age-specific HbA1c reference percentiles evaluated for all children were shown in Table 2 and Figure 3. Among all children, the 5th–95th percentiles of HbA1c (%) showed a “U” shape, decreasing first and then increasing. The age of 8 years old was the turning point. Considering that some indicators, such as BMI and blood lipids, differ between sexes, sex- and age-specific reference percentile curves and values of HbA1c (%) were constructed and are shown in Figures 3(b) and 3(c) and Table 2, respectively. Among boys, the 5th–95th percentiles of HbA1c (%) showed the same trend as those of all children; 8 years of age was the turning point among boys, while 9 years of age was the turning point among girls.

### 3.3. Influencing Factors Associated with HbA1c

**Figure 4:**
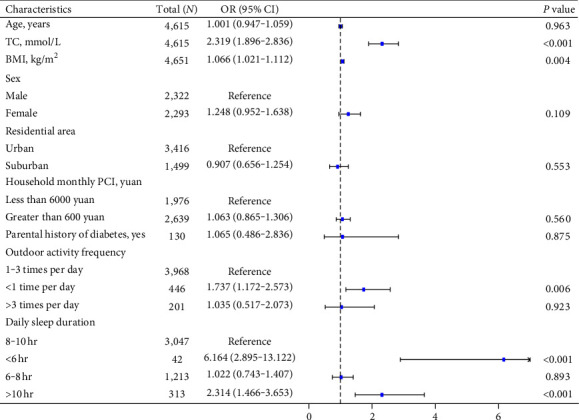
Influencing factors of the HbA1c levels over the 95th percentile value in the logistic regression model. BMI, body mass index; TC, total cholesterol; OR, odds ratio; CI, confidence interval. High HbA1c was defined as an HbA1c (%) value equal to or greater than the 95th percentile of the age- and sex-specific normal reference values derived from the study population.

All children were divided into two groups according to the 95th HbA1c percentile value. The baseline data of the two groups of children were compared, and the results are shown in Table 3. As shown in Figure 4, when assessing the 95th percentile of HbA1c (%) among the age-specific nomograms as the cutoff for high HbA1c, no statistically significant associations were detected between HbA1c levels and sex (*P* > 0.05). When assessing BMI, a one-unit increase in BMI (kg/m^2^) was associated with an increase in the risk of HbA1c (%) above the 95th percentile by approximately 0.066 times (*P*=0.004). We also clarified that higher HbA1c was significantly associated with higher TC levels. With a one-unit increase in serum TC (mmol/L), the risk of high HbA1c in children nearly increased by 1.319 times (*P* < 0.001). In addition, higher HbA1c levels were significantly associated with the frequency of outdoor activities. Compared to the one to three times per day group, the risk of a high HbA1c level in the less than one time group nearly increased by 0.737 times (*P*=0.006). Our results also indicated that daily sleep duration was significantly associated with higher HbA1c values. Compared to that in the 8–10-hr group, the risk of high HbA1c in the less than 6-hr group nearly increased by 5.164 times (*P* < 0.001), and the risk of high HbA1c in the above 10-hr group nearly increased by 2.314 times (*P* < 0.001).

### 3.4. Subgroup Analysis

The children were divided into a preschool-aged group (3–6 years old) and the school-aged group (7–12 years old). The results of univariable analysis were listed in *Supplementary table 3*.

**Figure 5:**
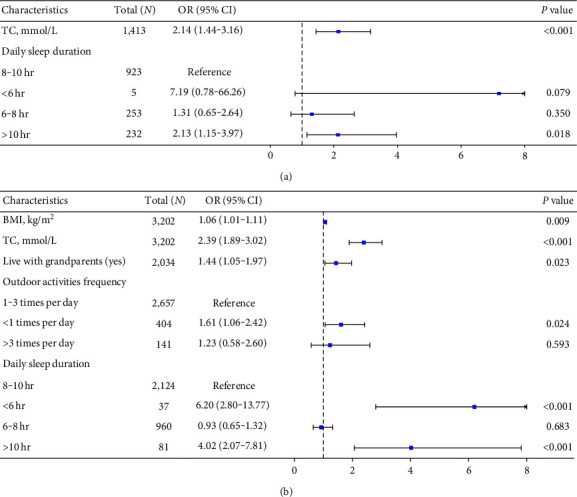
Influencing factors of the HbA1c levels over the 95th percentile value among study participants in two age subgroups in the logistic regression model: (a) 3–5 years old and (b) 6–12 years old.

The forest plot based on multivariable logistic regression analysis showed that in the preschool-aged group, significant factors influencing HbA1c level included TC level (OR = 2.15, 95% CI, 1.45–3.20, *P* < 0.001) and sleep duration ≥10 hr per day (OR = 2.13, 95% CI, 1.14–3.98, *P*=0.018) compared to 8–10 hr per day, and in the school-aged group, significant factors influencing HbA1c level included BMI (OR = 1.06, 95% CI, 1.01–1.11, *P*=0.009), TC (OR = 2.39, 95% CI, 1.89–3.02, *P* < 0.001), living with grandparents (OR = 1.44, 95% CI, 1.05–1.97, *P*=0.023), and frequency of outdoor activities less than once per day (OR = 1.61, 95% CI, 1.06–2.42, *P*=0.024) compared to one to three times per day, sleep duration <6 hr per day (OR = 6.20, 95% CI, 2.80–13.77, *P* < 0.001), and sleep duration ≥10 hr per day (OR = 4.02, 95% CI, 2.07–7.81, *P* < 0.001) compared to 8–10 hr per day (Figure 5). The results of subgroup analyses divided by typical pubertal age were consistent with the conclusions, TC; daily sleep duration remained a significant factor influencing A1C levels, that elevated A1C levels at younger ages may be associated with a family history of diabetes and outdoor activities, and that elevated A1C levels in the older group were associated with habits such as dine out and extracurricular lessons. The results of univariable analysis and multivariable logistic regression analysis were listed in *Supplementary table 4* and *Supplementary figure 5*.

## 4. Discussion

To our knowledge, it is one of the few studies that investigated the normative HbA1c levels among children. This study was designed to determine sex- and age-specific percentiles of capillary HbA1c based on Chinese healthy children aged 3–12 years, as well as potential risk factors associated with abnormal HbA1c.

We confirmed that the overall mean capillary HbA1c value was 5.3% (35.2 mmol/mol). Specifically, the mean capillary HbA1c value was 5.5% among preschoolers (3–5 years old) and 5.3% among school children (6–12 years old), respectively. Capillary HbA1c level in our research was higher than that in American children. Pettitt et al. [18] reported that the overall mean (SD) HbA1c value was 4.8% (range 3.4%–5.7%) among a total of 400 school children aged 11–13 years [18], and it was also higher than the intravenous HbA1c level (4.9%, range 3.5%–6.0%.) in Dutch children aged 8–9 years [19]. Differences in ethnicity and race [20], carbohydrate intake [21], and variation in red blood cell turnover [22] may explain the difference in the reported mean HbA1c value. Shanghai, situated in eastern China, stands as the largest economic center of the nation. The burgeoning economy of Shanghai has facilitated the availability of a wide array of calorie-dense and high-sugar foods coupled with a sedentary lifestyle, contributing to the observed rise in children and adolescent obesity rates. This phenomenon has substantial implications for metabolic health, which may also have led to higher HbA1c levels in children.

Our findings build upon previous studies that have investigated the percentile distribution of HbA1c across populations of varying ages and racial/ethnic backgrounds. For instance, in a small Caucasian population of 452 children and adolescents aged 6 months to 18 years, the reference interval of HbA1c from the 2.5th to 97.5th percentile ranged from 3.7% to 4.7% [23]. However, the limited sample size of this study constrained the precision of the estimates. The third National Health and Nutrition Examination Survey (NHANES III) collected HbA1c data from 7,968 American youths aged 5–24 years and reported a reference range of 4.5%–5.5% for the 10th to 95th percentiles [24]. However, the data were collected from 1988 to 1994, potentially limiting their relevance to the current context of the global epidemic of childhood and adolescent diabetes.

Our study did not detect statistically significant differences in HbA1c levels between boys and girls, which was consistent with a previous study [18], while Jansen et al. [19] reported that boys had a higher HbA1c than girls in a study population consisting solely of children aged 8–9 years. One plausible explanation could be that the inclusion of children across a broad age range may have mitigated the impact of pubertal hormonal fluctuations on HbA1c levels, thereby minimizing gender-related differences.

HbA1c levels was not nonuniformly and linearly correlated with age in our study. Explanations for the relationship between HbA1c level and age are rare, particularly concerning children. Rhee et al. [25] pointed that glucose levels increase significantly with age because of age-related impairment to glucose handling. Insulin sensitivity and insulin secretion both exhibit a decline with age, as evidenced by numerous studies in the literature, aligning with the observed rise in diabetes prevalence associated with aging [26]. As age increases, both glucose metabolism impairs and insulin action decreases. Another possible influencing factor was the onset of puberty; according to previous studies [27, 28], hormones such as growth hormones and sex hormones may increase insulin resistance, and the A1C level was elevated before the onset of puberty in both sexes, but it remains unclear to what extent this elevation is due to the onset of puberty. Further investigation into pathophysiological changes occurring with increasing age in childhood is recommended to gain a more comprehensive understanding of the progression of HbA1c levels over time.

We observed higher HbA1c levels in children with greater BMI, aligning with previous findings in Korean children [21] and other research conducted with adults [29]. Overweight and obesity serve as significant risk factors for the onset and progression of type 2 diabetes mellitus (T2DM). Studies have demonstrated a notably high prevalence of impaired glucose tolerance in obese children and adolescents [30], with insulin resistance being a related risk factor for impaired glucose tolerance, ultimately leading to elevated HbA1c levels. Physical activity is widely recognized as a lifestyle factor that reduces obesity, and the frequency of outdoor activities was found to be associated with HbA1c values in our study. Physical activity leads to glucose consumption, and individuals maintain a high level of energy expenditure for several hours after exercise. Our findings suggest that engaging in outdoor activities less than once per day increases the risk of having high HbA1c levels compared to a frequency of one to three times of outdoor activities, consistent with previous studies indicating a moderate impact of physical activity on HbA1c levels [31]. Moreover, our study revealed increasing HbA1c levels with increasing TC in children, which consists with the findings of Fu [32] and Xu et al. [33]. Lipid metabolism disorders have been observed in Chinese children with T2DM, and controlling intensive glucose has been shown to significantly decrease TC concentrations [34]. Thus, the rise in TC levels may be highly correlated with the increase in HbA1c levels. Furthermore, sleep duration was an additional factor related to HbA1c values in our study. The impact of sleep duration on insulin resistance and T2DM involves multiple pathways. We found that less than 6 hr or more than 10 hr of sleep increase the risk of having higher HbA1c levels, consistent with previous research [35]. Sleep is divided into four stages. During the third stage, REM (rapid eye movement) sleep, parasympathetic activity increases [36], which stimulates glucose-induced insulin secretion [37], ensuring the stability of blood sugar and glycosylated hemoglobin. A reduction in REM sleep duration leads to decreased insulin secretion. Furthermore, reduced sleep duration is also associated with secretion of leptin and growth hormone. Decreased leptin leads to increased hunger and appetite, especially for energy-dense foods, resulting in a higher likelihood of insulin resistance.

This study had some limitations. First, percentiles of HbA1c were derived from the Chinese population and need to be cautiously extrapolated to individuals of other races and ethnicities. Our study was conducted in Shanghai, an international metropolis in China, where westernized diets and lifestyles are widely adopted among young generations. Such a data source surely helps the generalization of HbA1c percentiles, but racial and ethnic variations in HbA1c percentiles might be overlooked. Second, selection bias may have occurred, 11% of eligible children refused to participate or failed to provide a blood sample which could have impacted our results. Finally, we did not investigate the typical characteristics of the onset of puberty, which may influence the association of risk factors with A1C levels given that puberty may increase insulin resistance and lead to a rise in A1C [38]. However, we performed sensitivity analyses using 10 years as the typical age of puberty onset, and the results were similar to the main results, suggesting robustness of the results. Studies with larger samples are still needed to validate the distribution of these percentiles and the association of associated risk factors with puberty.

## 5. Conclusions

This study enhanced our understanding of factors for HbA1c levels among children. Our results showed that BMI, TC, frequency of outdoor activities, and daily sleep duration were significantly associated with higher HbA1c levels. The age-specific percentiles of capillary HbA1c were also reported in children aged 3–12 years old. Further prospective longitudinal studies are suggested to elucidate using capillary HbA1c to identify diabetes or prediabetes at an early stage.

## Figures and Tables

**Table 1:** Characteristics of the subjects aged 3–12 years in Shanghai, China, 2018–2019.

Characteristic	All ages (*n* = 4,615)	3–5 years of age (*n* = 1,413)	6–12 years of age (*n* = 3,202)
Sociodemographic feature			
Sex, *n* (%)			
Boys	2,322 (50.3)	711 (50.3)	1,611 (50.3)
Girls	2,293 (49.7)	702 (49.7)	1,591 (49.7)
Age, median (IQR), years	7.3 (4.7)	3.9 (1.3)	8.9 (3.2)
Ethnicity (Han Chinese), *n* (*%*)	4,536 (98.3)	1,390 (98.4)	3,146 (98.3)
Residential area, *n* (*%*)			
Urban	3,416 (74.0)	907 (64.2)	2,509 (78.4)
Rural	1,499 (26.0)	506 (35.8)	693 (21.6)
Household monthly PCI, *n* (*%*)			
Less than 6,000 yuan	1,976 (42.8)	516 (36.5)	1,460 (45.6)
Greater than 6,000 yuan	2,639 (57.2)	897 (63.5)	1,742 (54.4)
Gestation, *n* (%)			
Premature	394 (8.5)	99 (7.0)	295 (9.2)
Full term	4,000 (86.7)	1,217 (86.1)	2,783 (86.9)
Postmature	221 (4.8)	97 (6.9)	124 (3.9)
Birthweight, *n* (%)			
Low birthweight	163 (3.5)	45 (3.2)	118 (3.7)
Normal birthweight	4,076 (88.3)	1,262 (89.3)	2,814 (88.3)
Macrosomia	376 (8.1)	106 (7.5)	270 (8.1)
Physical examination feature, *n* (%)			
Height, median (IQR), (cm)	127.0 (26.4)	108.0 (11.5)	134.0 (28.0)
Weight, median (IQR), (kg)	26.1 (15.0)	18.0 (4.1)	30.0 (13.6)
BMI, median (IQR), (kg/m^2^)	16.3 (3.4)	15.6 (1.3)	16.7 (4.0)
Weight status, *n* (*%*)			
Normal	3,556 (77.1)	1,143 (80.9)	2,413 (75.4)
Overweight	722 (15.6)	144 (10.2)	578 (18.1)
Obesity	337 (7.3)	126 (8.9)	211 (6.6)
Waist, median (IQR), (cm)	57.0 (12.0)	52.0 (6.0)	60.0 (11.0)
Hip, median (IQR), (cm)	67.0 (14.0)	60.0 (7.7)	71.0 (13.0)
Waist–hip ratio, median (IQR)	0.9 (0.1)	0.9 (0.1)	0.8 (0.1)
Laboratory indicators			
TC, median (IQR), (mmol/L)	3.8 (3.3, 4.4)	3.8 (3.4, 4.4)	3.8 (3.3, 4.4)
HDL, median (IQR), (mmol/L)	1.4 (1.3, 1.7)	1.4 (1.2, 1.6)	1.5 (1.3, 1.7)
TG, median (IQR), (mmol/L)	2.8 (2.7, 3.1)	2.9 (2.7, 3.1)	2.8 (2.7, 3.1)
LDL, median (IQR), (mmol/L)	1.4 (0.8, 2.0)	1.3 (0.8, 2.0)	1.4 (0.8, 2.0)
HbA1c (%), mean (SD)	5.3 ± 0.5	5.5 ± 0.4	5.3 ± 0.5
Family medical history, *n* (%)			
Hypertension	401 (8.7)	86 (6.1)	315 (9.8)
Diabetes	130 (2.8)	35 (2.5)	95 (3.0)
Dyslipidemia	299 (6.5)	73 (5.2)	226 (7.1)
Obesity	720 (15.6)	150 (10.6)	570 (17.8)
Liver diseases	135 (2.9)	54 (3.8)	81 (2.5)
Behavioral features			
Diet habits, *n* (%)			
High-sugar	2,013 (43.6)	641 (45.4)	1,372 (42.8)
High-salt	664 (14.4)	167 (11.8)	497 (15.5)
Fattier	183 (4.0)	33 (2.3)	150 (4.7)
Balanced	1,755 (38.0)	572 (40.5)	1,183 (36.9)
Breakfast (yes), *n* (%)	4,581 (99.3)	1,390 (98.4)	3,191 (99.7)
Daily meals ratio, *n* (%)			
1 : 1:1	2,007 (43.5)	667 (47.2)	1,340 (41.8)
3 : 4:3	1,018 (22.1)	295 (20.9)	723 (22.6)
2 : 4:4	1,590 (34.5)	451 (31.9)	1,139 (35.6)
Dine out frequency, *n* (%)			
0–1 times/month	868 (18.8)	271 (19.2)	597 (18.6)
2–3 times/month	2,142 (46.4)	624 (44.2)	1,518 (47.4)
1 time/week	1,028 (22.3)	335 (23.7)	693 (21.6)
1–3 times/week	483 (10.5)	149 (10.5)	334 (10.4)
Over 3 times/week	94 (2.0)	34 (2.4)	60 (1.9)
Between-meal nibbles, *n* (%)			
Never	89 (1.9)	38 (2.7)	51 (1.6)
Sometimes	2,994 (64.9)	909 (64.3)	2,085 (65.1)
Often	1,228 (26.6)	349 (34.7)	879 (27.5)
Everyday	304 (6.6)	117 (8.3)	187 (5.8)
Outdoor activities frequency (times per day)			
<1	446 (9.7)	42 (3.0)	404 (12.6)
1–3	3,968 (86.0)	1,311 (92.8)	2,657 (83.0)
>3	201 (4.4)	60 (4.2)	141 (4.4)
Outdoor activities time (min each time), *n* (%)			
<15	351 (7.6)	80 (5.7)	271 (8.5)
15–30	2,110 (45.7)	589 (41.7)	1,521 (47.5)
30–60	1,567 (34.0)	543 (38.4)	1,024 (32.0)
≥60	587 (12.7)	201 (14.2)	386 (12.1)
Outdoor activity intensity, *n* (%)			
High	1,915 (41.5)	527 (37.3)	1,388 (43.3)
Middle	2,616 (56.7)	865 (61.2)	1,751 (54.7)
Low	84 (1.8)	21 (1.5)	63 (2.0)
Sleep duration (hr per day), *n* (%)			
<6	42 (0.9)	5 (0.4)	37 (1.2)
6–8	1,213 (26.3)	253 (17.9)	960 (30.0)
8–10	3,047 (66.0)	923 (65.3)	2,124 (66.3)
≥10	313 (6.8)	232 (16.4)	81 (2.5)
Sleep time, *n* (%)			
Before 8 p.m.	65 (1.4)	26 (1.8)	39 (1.2)
8–10 p.m.	3,897 (84.4)	1,105 (78.2)	2,792 (87.2)
10–12 p.m.	643 (13.9)	274 (19.4)	369 (11.5)
After 12 p.m.	10 (0.2)	8 (0.8)	2 (0.1)
Sleep quality, *n* (%)			
Good	3,306 (71.6)	867 (61.4)	2,439 (76.2)
General	633 (13.7)	179 (12.7)	454 (14.2)
Poor	676 (14.6)	367 (26.0)	309 (9.7)
Nap (yes)	2,178 (47.2)	1,266 (89.6)	912 (28.5)
Passive smoking (yes)	315 (6.8)	80 (5.7)	235 (7.3)
Study time (hr per day), *n* (%)			
<8	2,697 (58.4)	1,236 (87.5)	1,461 (45.6)
8–10	1,568 (34.0)	157 (11.1)	1,411 (44.1)
10–12	305 (6.6)	15 (1.1)	290 (9.1)
≥12	45 (1.0)	5 (0.4)	40 (1.2)
Extracurricular lessons (days per week), *n* (%)			
Less 1 day	3,447 (74.7)	1,193 (84.4)	2,254 (70.4)
1 day	1,108 (24.0)	207 (14.6)	901 (28.1)
2 days	60 (1.3)	13 (0.9)	47 (1.5)
Digital products usage time (hr per day), *n* (%)			
<2	1,771 (85.2)	312 (81.5)	1,459 (86.0)
2–4	278 (13.4)	62 (16.2)	216 (12.7)
4–6	26 (1.3)	8 (2.1)	18 (1.1)
≥6	4 (0.2)	1 (0.3)	3 (0.2)

*Note*: The continuous and categorical variables were reported as mean ± SD/median (IQR) and count with column percentage, respectively. IQR, interquartile range; SD, standard deviation; PCI, per capita income; BMI, body mass index; TG, triglyceride; TC, total cholesterol; LDL-C, low-density lipoprotein cholesterol; HDL-C, high-density lipoprotein cholesterol. Household monthly PCI was dichotomized using the 2016 monthly average wage in Shanghai as the cutoff value.

**Table 2:** Sex- and age-specific reference percentile values of HbA1c (%) derived from all participants.

Age	P1	P3	P5	P10	P25	P50	P75	P90	P95	P97	P99
All children (years)											
3	4.7	4.9	5.0	5.2	5.4	5.6	5.8	6.1	6.2	6.3	6.5
4	4.4	4.7	4.8	5.0	5.2	5.5	5.8	6.0	6.2	6.3	6.6
5	4.1	4.5	4.6	4.8	5.1	5.4	5.7	5.9	6.1	6.3	6.6
6	4.0	4.3	4.5	4.7	5.0	5.3	5.6	5.8	6.1	6.2	6.5
7	3.9	4.3	4.4	4.7	4.9	5.2	5.5	5.8	6.0	6.1	6.5
8	3.8	4.3	4.4	4.6	4.9	5.2	5.5	5.7	6.0	6.1	6.5
9	3.9	4.3	4.5	4.7	5.0	5.2	5.5	5.7	5.9	6.1	6.5
10	3.9	4.4	4.5	4.7	5.0	5.3	5.5	5.8	6.0	6.1	6.5
11	3.9	4.4	4.5	4.8	5.1	5.3	5.6	5.9	6.1	6.3	6.7
12	3.8	4.4	4.6	4.8	5.1	5.4	5.7	6.0	6.2	6.4	6.9
Boys (years)											
3	4.5	4.8	4.9	5.1	5.4	5.6	5.9	6.2	6.3	6.5	6.8
4	4.3	4.7	4.8	5.0	5.3	5.5	5.8	6.0	6.2	6.4	6.7
5	4.2	4.5	4.7	4.9	5.1	5.4	5.7	5.9	6.1	6.2	6.6
6	4.1	4.4	4.6	4.8	5.0	5.3	5.5	5.8	6.0	6.2	6.5
7	4.0	4.4	4.5	4.7	5.0	5.2	5.5	5.7	5.9	6.1	6.5
8	3.9	4.3	4.5	4.7	5.0	5.2	5.5	5.7	5.9	6.1	6.5
9	3.9	4.3	4.5	4.7	5.0	5.2	5.5	5.7	6.0	6.1	6.5
10	3.9	4.3	4.5	4.7	5.0	5.3	5.5	5.8	6.0	6.2	6.6
11	3.9	4.4	4.5	4.8	5.1	5.3	5.6	5.9	6.1	6.3	6.8
12	3.9	4.4	4.6	4.9	5.2	5.4	5.7	6.0	6.2	6.4	7.0
Girls (years)											
3	4.6	4.8	5.0	5.1	5.4	5.6	5.9	6.1	6.2	6.4	6.6
4	4.4	4.7	4.8	5.0	5.2	5.5	5.7	6.0	6.1	6.3	6.5
5	4.3	4.5	4.7	4.9	5.1	5.4	5.6	5.9	6.1	6.2	6.4
6	4.1	4.4	4.6	4.8	5.0	5.3	5.6	5.8	6.0	6.1	6.4
7	4.0	4.3	4.5	4.7	5.0	5.2	5.5	5.8	6.0	6.1	6.4
8	3.9	4.3	4.4	4.6	4.9	5.2	5.5	5.7	5.9	6.1	6.4
9	3.8	4.2	4.4	4.6	4.9	5.2	5.5	5.8	6.0	6.1	6.5
10	3.8	4.2	4.4	4.7	5.0	5.3	5.5	5.8	6.0	6.2	6.6
11	3.8	4.3	4.5	4.7	5.0	5.3	5.6	5.9	6.2	6.3	6.8
12	3.8	4.3	4.5	4.8	5.1	5.4	5.7	6.0	6.3	6.5	7.0

**Table 3:** Baseline characteristics of study population divided by the 95th percentile of HbA1c (%) calculated with GAMLSS.

Variables	Normal level group (*n* = 4,377)	High level group (*n* = 238)	*P* value
Sociodemographic features			
Gender (male), *n* (%)	2,209 (50.5)	113 (47.5)	0.369
Age (years)	7.0 (5.0)	7.0 (4.75)	0.114
Race (Han), *n* (%)	4,299 (98.2)	237 (99.6)	0.115
Region (Urban), *n* (%)	3,232 (73.8)	184 (77.3)	0.234
Family members (≤4), *n* (%)	2,923 (66.8)	143 (60.1)	0.033
Monthly income (Chinese yuan), *n* (%)			0.127
≤3,000	412 (9.4)	15 (6.3)	—
3,000–6,000	1,458 (33.3)	91 (38.2)	—
>6,000	2,507 (57.3)	132 (55.5)	—
Gestation, *n* (%)			0.054
Premature	370 (8.5)	24 (10.1)	—
Full term	3,790 (86.6)	210 (88.2)	—
Postmature	217 (5.0)	4 (1.7)	—
Birthweight, *n* (%)			0.729
Low birthweight	156 (3.6)	7 (2.9)	—
Normal birthweight	3,862 (88.2)	214 (89.9)	—
Macrosomia	359 (8.2)	17 (7.1)	—
Physical examination features			
Height (cm)	127.0 (26.5)	130.0 (32)	0.012
Weight (kg)	26.0 (14.6)	28.0 (18.48)	0.003
BMI (kg/m^2^)	16.2 (3.3)	16.7 (4.9)	0.002
Weight status, *n* (%)			0.027
Normal	3,389 (77.4)	167 (70.2)	—
Overweight	676 (15.4)	46 (19.3)	—
Obesity	312 (7.1)	25 (10.5)	—
Waist (cm)	57.0 (12.0)	59.0 (14.0)	0.001
Hip (cm)	67.0 (14.0)	68.5 (15.0)	0.004
Waist–hip ratio	0.9 (0.1)	0.9 (0.1)	0.179
SBP (mmHg)	95.0 (19.0)	96.0 (22.0)	0.278
DBP (mmHg)	61.0 (14.0)	61.0 (16.0)	0.316
Laboratory indicators			
TC (mmol/L)	3.7 (1.0)	4.4 (0.9)	<.001
HDL (mmol/L)	1.4 (0.4)	1.4 (0.42)	0.740
TG (mmol/L)	2.8 (0.4)	2.8 (0.8)	0.262
LDL (mmol/L)	1.4 (1.2)	1.5 (1.3)	0.111
Family medical history, *n* (%)			
Hypertension	372 (8.5)	29 (12.2)	0.049
Diabetes	123 (2.8)	7 (2.9)	0.905
Dyslipidemia	287 (6.6)	12 (5.0)	0.355
Obesity	682 (15.6)	38 (16.0)	0.873
Liver diseases	127 (2.9)	8 (3.4)	0.682
Behavioral features, *n* (%)			
Diet habits			0.271
High-sugar	1,917 (43.8)	96 (40.3)	—
High-salt	636 (14.5)	28 (11.8)	—
Fattier	172 (3.9)	11 (4.6)	—
Balanced	1,652 (37.7)	103 (43.3)	—
Breakfast (yes), *n* (%)	4,344 (99.2)	237 (99.6)	0.558
Daily meals ratio, *n* (%)			0.671
1 : 1:1	1,910 (43.6)	97 (40.8)	—
3 : 4:3	964 (22.0)	54 (22.7)	—
2 : 4:4	1,503 (34.3)	87 (36.6)	—
Dine out frequency, *n* (%)			0.568
0–1 times/month	833 (19.0)	35 (14.7)	—
2–3 times/month	2,027 (46.3)	115 (48.3)	—
1 time/week	970 (22.2)	58 (24.4)	—
1–3 times/week	458 (10.5)	25 (10.5)	—
Over 3 times/week	89 (2.0)	5 (2.1)	—
Between-meal nibbles, *n* (%)			0.977
Never	84 (1.9)	5 (2.1)	—
Sometimes	2,837 (64.8)	157 (66.0)	—
Often	1,167 (26.7)	61 (25.6)	—
Everyday	289 (6.6)	15 (6.3)	—
Outdoor activities frequency (times per day), *n* (%)			0.001
<1	407 (9.3)	39 (16.4)	—
1–3	3,778 (86.3)	190 (79.8)	—
>3	192 (4.4)	9 (3.8)	—
Outdoor activities time (minutes each time), *n* (%)			0.869
<15	331 (7.6)	20 (8.4)	—
15–30	2,006 (45.8)	104 (43.7)	—
30–60	1,482 (33.9)	85 (35.7)	—
≥60	558 (12.7)	29 (12.2)	—
Outdoor activities intensity, *n* (%)			0.509
High	1,815 (41.5)	100 (42.0)	—
Middle	2,480 (56.7)	136 (57.1)	—
Low	82 (1.9)	2 (0.8)	—
Sleep duration (hr per day), *n* (%)			<0.001
<6	31 (0.7)	11 (4.6)	—
6–8	1,153 (26.3)	60 (25.2)	—
8–10	2,908 (66.4)	139 (58.4)	—
≥10	285 (6.5)	28 (11.8)	—
Sleep time, *n* (%)			0.833
Before 8 p.m.	60 (1.4)	4 (1.7)	—
8–10 p.m.	3,695 (84.4)	203 (85.3)	—
10–12 p.m.	612 (14.0)	31 (13.0)	—
After 12 p.m.	10 (0.2)	0 (0.0)	—
Sleep quality, *n* (%)			0.222
Good	3,142 (71.8)	164 (68.9)	—
General	603 (13.8)	30 (12.6)	—
Poor	632 (14.4)	44 (18.5)	—
Nap (yes), *n* (%)	2,062 (47.1)	116 (48.7)	0.624
Passive smoking (yes), *n* (%)	296 (6.8)	19 (8.0)	0.467
Study time (hr per day), *n* (%)			0.339
<8	2,556 (58.4)	141 (59.2)	—
8–10	1,482 (33.9)	86 (36.1)	—
10–12	295 (6.7)	10 (4.2)	—
≥12	44 (1.0)	1 (0.4)	—
Extracurricular lessons (days per week), *n* (%)			0.360
Less 1 day	3,263 (74.5)	184 (77.3)	—
1 day	1,055 (24.1)	53 (22.3)	—
2 days	59 (1.3)	1 (0.4)	—
Digital products usage time (hr per day), *n* (%)			0.467
<2	1,636 (84.9)	135 (89.4)	—
2–4	263 (13.6)	15 (9.9)	—
4–6	25 (1.3)	1 (0.7)	—
≥6	4 (0.2)	0 (0.0)	—

*Note*: The continuous and categorical variables were reported as mean ± SD or median (IQR) and count with column percentage, respectively. SD, standard deviation; IQR, interquartile range; PCI, per capita income; BMI, body mass index; TG, triglyceride; TC, total cholesterol; LDL-C, low-density lipoprotein cholesterol; HDL-C, high-density lipoprotein cholesterol. Household monthly PCI was dichotomized using the 2016 monthly average wage in Shanghai as the cutoff value.

## Data Availability

Anonymized data collected for the study and a data dictionary will be made available to other researchers following the approval of a study proposal by Lijuan Zhang (zhangxiaoyi@tongji.edu.cn) for 5 years after publication. The study protocol, statistical analysis plan, and informed consent forms are also available from Jue Li.

## Supplementary Materials

Supplementary 1 Table S1: comparison of sociodemographic characteristics between included and excluded participants.

Supplementary 2 Table S2: anthropometric and HbA1c distribution of study population by sex and age, Shanghai, China, 2018–2019.

Supplementary 3 Table S3: baseline characteristics of two subgroups (3–5 years of age 6–12 years of age) divided by the 95th percentile of HbA1c (%) from study population calculated with GAMLSS.

Supplementary 4 Table S4: baseline characteristics of two subgroups (3–9 years of age 10–12 years of age) and divided by the 95th percentile of HbA1c (%) from study population calculated with GAMLSS.

Supplementary 5 Figure S1: influencing factors of the HbA1c levels over the 95th percentile value of two subgroups (3–9 years old and 10–12 years old children) in the logistic regression model.
