# Supplementary material for: Capillary Glycated Hemoglobin A1c Percentiles and the Risk Factors Associated with Abnormal HbA1c among Chinese Children Aged 3–12 Years
Source: Pediatr Diabetes. 2024 Jul 29;2024:8333590. doi: 10.1155/2024/8333590 (PMC12017142; doi:10.1155/2024/8333590)
Supplement: Supplementary 1 — Table S1: comparison of sociodemographic characteristics between included and excluded participants. [file 8333590.f1.docx]

|  | Included  (*n* = 4,615) | Excluded  (*n* = 185) | *p* value |
| --- | --- | --- | --- |
| Sex, *n* (%) |  |  | 0.782 |
| Boys | 2322 (50.3) | 95 (51.4) |  |
| Girls | 2293 (49.7) | 90 (48.6) |  |
| Age, median (IQR), years | 7.3 (4.7) | 7.5 (4.9) | 0.571 |
| Ethnicity (Han Chinese), *n (%)* | 4536 (98.3) | 182 (98.4) | 0.844 |
| Residential area, *n (%)* |  |  | 0.591 |
| Urban | 3416 (74.0) | 132 (71.4) |  |
| Rural | 1499 (26.0) | 53 (28.6) |  |
| Household monthly PCI, *n (%)* | |  | 0.201 |
| Less than 6000 yuan | 1976 (42.8) | 88 (47.6) |  |
| Greater than 6000 yuan | 2639 (57.2) | 97 (52.4) |  |

Table S1. Comparison of sociodemographic characteristics between included and excluded participants
