# Supplementary material for: Capillary Glycated Hemoglobin A1c Percentiles and the Risk Factors Associated with Abnormal HbA1c among Chinese Children Aged 3–12 Years
Source: Pediatr Diabetes. 2024 Jul 29;2024:8333590. doi: 10.1155/2024/8333590 (PMC12017142; doi:10.1155/2024/8333590)
Supplement: Supplementary 2 — Table S2: anthropometric and HbA1c distribution of study population by sex and age, Shanghai, China, 2018–2019. [file 8333590.f2.docx]

Table S2. Anthropometric and HbA1c distribution of study population by sex and age, Shanghai, China, 2018-2019

| Age | N | Height (cm) | Weight (kg) | BMI (kg/m2) | HbA1c (%) |
| --- | --- | --- | --- | --- | --- |
| Boys |  |  |  |  |  |
| 3 years | 201 | 102.8 ± 5.2 | 16.4 ± 2.5 | 15.5 ± 1.8 | 5.6 ± 0.4 |
| 4 years | 278 | 106.0 ± 5.4 | 19.0 ± 3.0 | 15.8 ± 1.8 | 5.6 ± 0.4 |
| 5 years | 232 | 113.5 ± 6.8 | 20.7 ± 4.1 | 16.0 ± 2.0 | 5.4 ± 0.5 |
| 6 years | 260 | 120.4 ± 5.1 | 23.8 ± 4.8 | 16.4 ± 2.6 | 5.3 ± 0.5 |
| 7 years | 269 | 125.2 ± 4.9 | 26.3 ± 5.5 | 16.6 ± 2.7 | 5.2 ± 0.5 |
| 8 years | 232 | 129.9 ± 5.6 | 29.7 ± 6.1 | 17.5 ± 2.8 | 5.2 ± 0.6 |
| 9 years | 205 | 135.3 ± 5.8 | 33.3 ± 7.1 | 18.1 ± 3.1 | 5.2 ± 0.4 |
| 10 years | 238 | 143.2 ± 7.4 | 40.3 ± 8.6 | 19.5 ± 2.8 | 5.3 ± 0.4 |
| 11 years | 200 | 149.6 ± 7.7 | 46.5 ± 11.5 | 20.6 ± 4.1 | 5.3 ± 0.6 |
| 12 years | 207 | 154.7 ± 7.3 | 49.5 ± 12.0 | 20.5 ± 3.8 | 5.5 ± 0.5 |
| ALL | 2322 | 128.8 ± 17.5 | 30.8 ± 13.3 | 17.7 ± 3.4 | 5.3 ± 0.5 |
| Girls |  |  |  |  |  |
| 3 years | 213 | 101.5 ± 5.6 | 16.2 ± 2.7 | 15.7 ± 1.6 | 5.6 ± 0.4 |
| 4 years | 248 | 104.9 ± 6.2 | 17.5 ± 2.6 | 15.6 ± 1.3 | 5.5 ± 0.5 |
| 5 years | 241 | 111.5 ± 5.3 | 19.0 ± 3.0 | 15.3 ± 1.4 | 5.3 ± 0.4 |
| 6 years | 206 | 118.1 ± 5.5 | 21.9 ± 4.1 | 15.6 ± 1.9 | 5.3 ± 0.4 |
| 7 years | 228 | 123.7 ± 6.4 | 24.2 ± 4.7 | 15.7 ± 2.0 | 5.3 ± 0.6 |
| 8 years | 204 | 127.9 ± 6.2 | 27.1 ± 5.0 | 16.4 ± 2.1 | 5.2 ± 0.5 |
| 9 years | 252 | 133.7 ± 6.7 | 29.7 ± 4.9 | 16.5 ± 2.0 | 5.2 ± 0.5 |
| 10 years | 268 | 141.4 ± 7.1 | 34.8 ± 7.2 | 17.3 ± 2.6 | 5.3 ± 0.5 |
| 11 years | 200 | 149.0 ± 6.8 | 40.9 ± 9.1 | 18.3 ± 3.4 | 5.4 ± 0.6 |
| 12 years | 233 | 154.1 ± 6.6 | 46.4 ± 9.7 | 19.4 ± 3.4 | 5.4 ± 0.5 |
| ALL | 2293 | 128.9 ± 18.3 | 29.1 ± 11.8 | 16.8 ± 2.8 | 5.3 ± 0.5 |

Anthropometric and HbA1c distribution were described using mean ± standard deviation.
