# Supplementary material for: Capillary Glycated Hemoglobin A1c Percentiles and the Risk Factors Associated with Abnormal HbA1c among Chinese Children Aged 3–12 Years
Source: Pediatr Diabetes. 2024 Jul 29;2024:8333590. doi: 10.1155/2024/8333590 (PMC12017142; doi:10.1155/2024/8333590)
Supplement: Supplementary 3 — Table S3: baseline characteristics of two subgroups (3–5 years of age 6–12 years of age) divided by the 95th percentile of HbA1c (%) from study population calculated with GAMLSS. [file 8333590.f3.docx]

Table S3 Baseline characteristics of two subgroups (3-5 years of age 6-12 years of age) divided by the 95th percentile of HbA1c (%) from study population calculated with GAMLSS.

| Variables | Preschool-age children | | |  | School-age children | | |
| --- | --- | --- | --- | --- | --- | --- | --- |
|  | Normal level group  (n = 1354) | High level group  (n = 59) | P |  | Normal level group  (n = 3023) | High level group  (n = 179) | P |
| Sociodemographic features | | | | | | | |
| Age | 4.00 (3.00 - 5.00) | 4.00 (0.50) | 0.534 |  | 9.00 (7.00 - 11.00) | 9.00 (4.00) | 0.914 |
| Gender (male), n (%) | 685 (50.59) | 26 (44.07) | 0.327 |  | 1524 (50.41) | 87 (48.60) | 0.638 |
| Race (Han), n (%) | 1332 (98.38) | 58 (98.31) | 1.000 |  | 2967 (98.15) | 179 (100.00) | 0.123 |
| Live with grandparents, n (%) | | | 0.785 |  |  |  | 0.019 |
| Yes | 988 (72.97) | 44 (74.58) |  |  | 1935 (64.01) | 99 (55.31) |  |
| No | 366 (27.03) | 15 (25.42) |  |  | 1088 (35.99) | 80 (44.69) |  |
| Monthly income (Chinese yuan), n (%) | | | 0.568 |  |  |  | 0.167 |
| ≤3000 | 94 (6.94) | 2 (3.39) |  |  | 318 (10.52) | 13 (7.26) |  |
| 3000~6000 | 402 (29.69) | 18 (30.51) |  |  | 1056 (34.93) | 73 (40.78) |  |
| >6000 | 858 (63.37) | 39 (66.10) |  |  | 1649 (54.55) | 93 (51.96) |  |
| Birthweight, n (%) | | | 0.573 |  |  |  | 0.533 |
| Low birthweight | 42 (3.10) | 3 (5.08) |  |  | 114 (3.77) | 4 (2.23) |  |
| Normal birthweight | 1209 (89.29) | 53 (89.83) |  |  | 2653 (87.76) | 161 (89.94) |  |
| Macrosomia | 103 (7.61) | 3 (5.08) |  |  | 256 (8.47) | 14 (7.82) |  |
| Physical examination features | | |  |  |  |  |  |
| Height (cm) | 108.00 (11.20) | 108.00 (11.00) | 0.677 |  | 134.00 (19.00) | 135.00 (27.00) | 0.194 |
| Weight (Kg) | 18.00 (4.10) | 18.10 (4.10) | 0.583 |  | 30.00 (13.30) | 31.70 (19.75) | 0.024 |
| BMI (Kg/m^2^) | 15.62 (2.26) | 15.53 (2.75) | 0.778 |  | 17.14 (5.45) | 17.14 (5.45) | 0.005 |
| Waist circumference(cm) | 52.00 (6.00) | 52.00 (5.50) | 0.686 |  | 60.00 (11.00) | 61.20 (14.00) | 0.003 |
| Hip circumference(cm) | 60.00 (7.50) | 60.00 (7.50) | 0.549 |  | 71.00 (13.00) | 72.00 (15.85) | 0.005 |
| Waist-hip ratio | 0.87 (0.08) | 0.87 (0.08) | 0.288 |  | 0.85 (0.08) | 0.85 (0.07) | 0.286 |
| SBP (mmHg) | 89.00 (83.00 - 96.00) | 88.00 (11.50) | 0.105 |  | 99.00 (19.00) | 101.00 (18.00) | 0.158 |
| DBP (mmHg) | 58.00 (53.00 - 63.00) | 57.00 (11.00) | 0.675 |  | 63.00 (14.00) | 65.00 (14.00) | 0.335 |
| Laboratory indicators | | |  |  |  |  |  |
| TC (mmol/L) | 3.75 (0.95) | 4.29 (0.92) | <.001 |  | 3.72 (1.06) | 4.39 (0.90) | <.001 |
| HDL (mmol/L) | 1.40 (0.35) | 1.38 (0.31) | 0.982 |  | 1.46 (0.41) | 1.46 (0.47) | 0.805 |
| TG (mmol/L) | 2.85 (0.44) | 2.93 (0.41) | 0.464 |  | 2.84 (0.41) | 2.81 (0.38) | 0.095 |
| LDL (mmol/L) | 1.31 (1.13) | 1.35 (0.89) | 0.662 |  | 1.39 (1.22) | 1.55 (1.39) | 0.101 |
| Family medical history, n (%) | | | |  |  |  |  |
| Hypertension (yes) | 80 (5.91) | 6 (10.17) | 0.288 |  | 292 (9.66) | 23 (12.85) | 0.164 |
| Diabetes (yes) | 31 (2.29) | 4 (6.78) | 0.081 |  | 92 (3.04) | 3 (1.68) | 0.295 |
| Dyslipidemia (yes) | 69 (5.10) | 4 (6.78) | 0.786 |  | 218 (7.21) | 8 (4.47) | 0.164 |
| Obesity (yes) | 143 (10.56) | 7 (11.86) | 0.750 |  | 539 (17.83) | 31 (17.32) | 0.862 |
| Liver diseases (yes) | 53 (3.91) | 1 (1.69) | 0.601 |  | 74 (2.45) | 7 (3.91) | 0.334 |
| Behavioral features, n (%) | | | |  |  |  |  |
| Diet habits |  |  | 0.926 |  |  |  | 0.212 |
| High-sugar | 616 (45.49) | 25 (42.37) |  |  | 1301 (43.04) | 71 (39.66) |  |
| High-salt | 160 (11.82) | 7 (11.86) |  |  | 476 (15.75) | 21 (11.73) |  |
| Fattier | 31 (2.29) | 2 (3.39) |  |  | 141 (4.66) | 9 (5.03) |  |
| Balanced | 547 (40.40) | 25 (42.37) |  |  | 1105 (36.55) | 78 (43.58) |  |
| Breakfast , n (%) | | | 1.000 |  |  |  | 1.000 |
| Yes | 1332 (98.38) | 58 (98.31) |  |  | 3012 (99.64) | 179 (100.00) |  |
| No | 22 (1.62) | 1 (1.69) |  |  | 11 (0.36) | 0 (0.00) |  |
| Daily meals ratio, n (%) | | | 0.432 |  |  |  | 0.977 |
| 1:1:1 | 644 (47.56) | 23 (38.98) |  |  | 1266 (41.88) | 74 (41.34) |  |
| 3:4:3 | 281 (20.75) | 14 (23.73) |  |  | 683 (22.59) | 40 (22.35) |  |
| 2:4:4 | 429 (31.68) | 22 (37.29) |  |  | 1074 (35.53) | 65 (36.31) |  |
| Dine out frequency, n (%) | | | 0.782 |  |  |  | 0.392 |
| 0-1 times/month | 260 (19.20) | 11 (18.64) |  |  | 573 (18.95) | 24 (13.41) |  |
| 2-3 times/month | 602 (44.46) | 22 (37.29) |  |  | 1425 (47.14) | 93 (51.96) |  |
| 1 times/week | 319 (23.56) | 16 (27.12) |  |  | 651 (21.53) | 42 (23.46) |  |
| 1-3 times/week | 141 (10.41) | 8 (13.56) |  |  | 317 (10.49) | 17 (9.50) |  |
| Over 3 times/week | 32 (2.36) | 2 (3.39) |  |  | 57 (1.89) | 3 (1.68) |  |
| Between-meal nibbles, n (%) | | | 0.963 |  |  |  | 0.804 |
| Never | 37 (2.73) | 1 (1.69) |  |  | 47 (1.55) | 4 (2.23) |  |
| Sometimes | 872 (64.40) | 37 (62.71) |  |  | 1965 (65.00) | 120 (67.04) |  |
| Often | 333 (24.59) | 16 (27.12) |  |  | 834 (27.59) | 45 (25.14) |  |
| Everyday | 112 (8.27) | 5 (8.47) |  |  | 177 (5.86) | 10 (5.59) |  |
| Outdoor activities frequency (times per day), n (%) | | | 0.139 |  |  |  | 0.015 |
| ＜1 | 38 (2.81) | 4 (6.78) |  |  | 369 (12.21) | 35 (19.55) |  |
| 1-3 | 1257 (92.84) | 54 (91.53) |  |  | 2521 (83.39) | 136 (75.98) |  |
| >3 | 59 (4.36) | 1 (1.69) |  |  | 133 (4.40) | 8 (4.47) |  |
| Outdoor activities time (min each time), n (%) | | | 0.182 |  |  |  | 0.536 |
| ＜15 | 73 (5.39) | 7 (11.86) |  |  | 258 (8.53) | 13 (7.26) |  |
| 15-30 | 565 (41.73) | 24 (40.68) |  |  | 1441 (47.67) | 80 (44.69) |  |
| 30-60 | 524 (38.70) | 19 (32.20) |  |  | 958 (31.69) | 66 (36.87) |  |
| ≥60 | 192 (14.18) | 9 (15.25) |  |  | 366 (12.11) | 20 (11.17) |  |
| Sleep duration (hours per day), n (%) | | | 0.031 |  |  |  | <.001 |
| ＜6 | 4 (0.30) | 1 (1.69) |  |  | 27 (0.89) | 10 (5.59) |  |
| 6-8 | 242 (17.87) | 11 (18.64) |  |  | 911 (30.14) | 49 (27.37) |  |
| 8-10 | 892 (65.88) | 31 (52.54) |  |  | 2016 (66.69) | 108 (60.34) |  |
| ≥10 | 216 (15.95) | 16 (27.12) |  |  | 69 (2.28) | 12 (6.70) |  |
| Sleep time, n (%) | | | 0.702 |  |  |  | 0.197 |
| Before 8 p.m. | 26 (1.92) | 0 (0.00) |  |  | 34 (1.12) | 5 (2.79) |  |
| 8-10 p.m. | 1060 (78.29) | 45 (76.27) |  |  | 2635 (87.17) | 157 (87.71) |  |
| 10-12 p.m. | 260 (19.20) | 14 (23.73) |  |  | 352 (11.64) | 17 (9.50) |  |
| After 12 p.m. | 8 (0.59) | 0 (0.00) |  |  | 2 (0.07) | 0 (0.00) |  |
| Sleep quality, n (%) | | | 0.074 |  |  |  | 0.092 |
| Good | 829 (61.23) | 38 (64.41) |  |  | 2313 (76.51) | 126 (70.39) |  |
| General | 177 (13.07) | 2 (3.39) |  |  | 426 (14.09) | 28 (15.64) |  |
| Poor | 348 (25.70) | 19 (32.20) |  |  | 284 (9.39) | 25 (13.97) |  |
| Passive smoking (yes), n (%) | | | 0.102 |  |  |  | 0.084 |
| 1 | 80 (5.91) | 0 (0.00) |  |  | 216 (7.15) | 19 (10.61) |  |
| 2 | 1274 (94.09) | 59 (100.00) |  |  | 2807 (92.85) | 160 (89.39) |  |
| Study time (hours per day), n (%) | | | 0.862 |  |  |  | 0.299 |
| ＜8 | 1182 (87.30) | 54 (91.53) |  |  | 1374 (45.45) | 87 (48.60) |  |
| 8-10 | 152 (11.23) | 5 (8.47) |  |  | 1330 (44.00) | 81 (45.25) |  |
| 10-12 | 15 (1.11) | 0 (0.00) |  |  | 280 (9.26) | 10 (5.59) |  |
| ≥12 | 5 (0.37) | 0 (0.00) |  |  | 39 (1.29) | 1 (0.56) |  |
| Extracurricular (days per week), n (%) | | | 0.351 |  |  |  | 0.140 |
| Less one day | 1146 (84.64) | 47 (79.66) |  |  | 2117 (70.03) | 137 (76.54) |  |
| One day | 195 (14.40) | 12 (20.34) |  |  | 860 (28.45) | 41 (22.91) |  |
| Two days | 13 (0.96) | 0 (0.00) |  |  | 46 (1.52) | 1 (0.56) |  |
| Digital products usage time (hours per day), n (%) | | | 0.350 |  |  |  | 0.488 |
| ＜2 | 297 (81.37) | 15 (83.33) |  |  | 1339 (85.67) | 120 (90.23) |  |
| 2-4 | 60 (16.44) | 2 (11.11) |  |  | 203 (12.99) | 13 (9.77) |  |
| 4-6 | 7 (1.92) | 1 (5.56) |  |  | 18 (1.15) | 0 (0.00) |  |
| ≥6 | 1 (0.27) | 0 (0.00) |  |  | 3 (0.19) | 0 (0.00) |  |
