# Supplementary material for: Capillary Glycated Hemoglobin A1c Percentiles and the Risk Factors Associated with Abnormal HbA1c among Chinese Children Aged 3–12 Years
Source: Pediatr Diabetes. 2024 Jul 29;2024:8333590. doi: 10.1155/2024/8333590 (PMC12017142; doi:10.1155/2024/8333590)
Supplement: Supplementary 4 — Table S4: baseline characteristics of two subgroups (3–9 years of age 10–12 years of age) and divided by the 95th percentile of HbA1c (%) from study population calculated with GAMLSS. [file 8333590.f4.docx]

Table S4 Baseline characteristics of two subgroups (3-9 years of age 10-12 years of age) and divided by the 95th percentile of HbA1c (%) from study population calculated with GAMLSS.

| Variables | 3-9 years of age | | |  | 10-12 years of age | | |
| --- | --- | --- | --- | --- | --- | --- | --- |
|  | Normal level group  (n = 3429) | High level group  (n = 162) | *P* |  | Normal level group  (n = 948) | High level group  (n = 76) | *P* |
| Sociodemographic features | | | | | | | |
| Age,median (IQR), years | 5.30 (0.60) | 6.30 (0.40) | <0.001 |  | 11.30 (1.28) | 11.24 (1.04) | 0.710 |
| Gender (male), n (%) | 1753 (51.12) | 81 (50.00) | 0.780 |  | 456 (48.10) | 32 (42.11) | 0.314 |
| Race (Han), n (%) | 3364 (98.10) | 161 (99.38) | 0.376 |  | 935 (98.63) | 76 (100.00) | 0.615 |
| Live with grandparents, n (%) | | | 0.181 |  |  |  | 0.248 |
| Yes | 2372 (69.17) | 104 (64.20) |  |  | 551 (58.12) | 39 (51.32) |  |
| No | 1057 (30.83) | 58 (35.80) |  |  | 397 (41.88) | 37 (48.68) |  |
| Monthly income (Chinese yuan), n (%) | | | 0.809 |  |  |  | 0.230 |
| ≤6000 | 1451 (42.32) | 67 (41.36) |  |  | 419 (44.20) | 39 (51.32) |  |
| >6000 | 1978 (57.68) | 95 (58.64) |  |  | 529 (55.80) | 37 (48.68) |  |
| Birthweight, n (%) | | | 0.278 |  |  |  | 0.528 |
| Low birthweight | 130 (3.62) | 127 (3.70) |  |  | 29 (3.06) | 4 (5.26) |  |
| Normal birthweight | 3168 (88.22) | 3019 (88.04) |  |  | 843 (88.92) | 65 (85.53) |  |
| Macrosomia | 293 (8.16) | 283 (8.25) |  |  | 76 (8.02) | 7 (9.21) |  |
| Physical examination features | | |  |  |  |  |  |
| Height (cm) | 121.50 (20.80) | 120.25 (17.75) | 0.503 |  | 149.00 (12.00) | 152.00 (6.63) | 0.032 |
| Weight (Kg) | 23.20 (10.10) | 23.50 (8.96) | 0.785 |  | 40.60 (14.60) | 47.00 (16.20) | <.001 |
| BMI (Kg/m^2^) | 15.92 (2.68) | 16.18 (2.62) | 0.890 |  | 18.33 (5.22) | 21.25 (6.59) | <.001 |
| Waist circumference(cm) | 55.00 (9.00) | 55.00 (9.00) | 0.758 |  | 66.00 (12.00) | 70.50 (13.00) | <.001 |
| Hip circumference(cm) | 65.00 (10.00) | 65.00 (10.00) | 0.811 |  | 80.00 (11.00) | 85.00 (13.00) | <.001 |
| Waist-hip ratio | 0.86 (0.07) | 0.85 (0.06) | 0.418 |  | 0.83 (0.08) | 0.87 (0.08) | <.001 |
| SBP (mmHg) | 93.00 (15.00) | 93.00 (15.75) | 0.105 |  | 106.00 (17.00) | 108.00 (15.25) | 0.418 |
| DBP (mmHg) | 60.00 (12.00) | 59.50 (14.75) | 0.675 |  | 66.00 (14.00) | 69.00 (16.00) | 0.038 |
| Laboratory indicators | | |  |  |  |  |  |
| TC (mmol/L) | 3.73 (0.99) | 4.37 (0.86) | <.001 |  | 3.71 (1.16) | 4.59 (1.13) | 0.002 |
| HDL (mmol/L) | 1.44 (0.40) | 1.45 (0.39) | 0.559 |  | 1.44 (0.37) | 1.43 (0.46) | 0.839 |
| TG (mmol/L) | 2.84 (0.41) | 2.83 (0.59) | 0.308 |  | 2.84 (0.41) | 2.82 (0.39) | 0.627 |
| LDL (mmol/L) | 1.34 (1.16) | 1.52 (1.10) | 0.040 |  | 1.45 (1.30) | 1.43 (1.50) | 0.969 |
| Family medical history, (yes), n (%) | | | |  |  |  |  |
| Hypertension | 268 (7.82) | 20 (12.35) | 0.038 |  | 104 (10.97) | 9 (11.84) | 0.815 |
| Diabetes | 99 (2.89) | 7 (4.32) | 0.414 |  | 24 (2.53) | 0 (0.00) | 0.313 |
| Dyslipidemia | 207 (6.04) | 8 (4.94) | 0.565 |  | 80 (8.44) | 4 (5.26) | 0.332 |
| Obesity | 546 (15.92) | 26 (16.05) | 0.966 |  | 136 (14.35) | 12 (15.79) | 0.731 |
| Liver diseases | 102 (2.97) | 3 (1.85) | 0.555 |  | 25 (2.64) | 5 (6.58) | 0.108 |
| Behavioral features, n (%) | | | |  |  |  |  |
| Diet habits |  |  | 0.610 |  |  |  | 0.425 |
| High-sugar | 1537 (44.82) | 68 (41.98) |  |  | 380 (40.08) | 28 (36.84) |  |
| High-salt | 457 (13.33) | 18 (11.11) |  |  | 179 (18.88) | 10 (13.16) |  |
| Fattier | 120 (3.50) | 6 (3.70) |  |  | 52 (5.49) | 5 (6.58) |  |
| Balanced | 1315 (38.35) | 70 (43.21) |  |  | 337 (35.55) | 33 (43.42) |  |
| Breakfast , n (%) | | | 1.000 |  |  |  | 1.000 |
| Yes | 3399 (99.13) | 161 (99.38) |  |  | 945 (99.68) | 76 (100.00) |  |
| No | 30 (0.87) | 1 (0.62) |  |  | 3 (0.32) | 0 (0.00) |  |
| Daily meals ratio, n (%) | | | 0.499 |  |  |  | 0.869 |
| 1:1:1 | 1535 (44.77) | 65 (40.12) |  |  | 375 (39.56) | 32 (42.11) |  |
| 3:4:3 | 782 (22.81) | 41 (25.31) |  |  | 182 (19.20) | 13 (17.11) |  |
| 2:4:4 | 1112 (32.43) | 56 (34.57) |  |  | 391 (41.24) | 31 (40.79) |  |
| Dine out frequency, n (%) | | | 0.119 |  |  |  | 0.009 |
| 0-1 times/month | 590 (17.21) | 22 (13.58) |  |  | 243 (25.63) | 13 (17.11) |  |
| 2-3 times/month | 1602 (46.72) | 65 (40.12) |  |  | 425 (44.83) | 50 (65.79) |  |
| 1 times/week | 798 (23.27) | 48 (29.63) |  |  | 172 (18.14) | 10 (13.16) |  |
| 1-3 times/week | 372 (10.85) | 22 (13.58) |  |  | 86 (9.07) | 3 (3.95) |  |
| Over 3 times/week | 67 (1.95) | 5 (3.09) |  |  | 22 (2.32) | 0 (0.00) |  |
| Between-meal nibbles, n (%) | | | 0.993 |  |  |  | 0.591 |
| Never | 73 (2.13) | 4 (2.47) |  |  | 11 (1.16) | 1 (1.32) |  |
| Sometimes | 2314 (67.48) | 109 (67.28) |  |  | 523 (55.17) | 48 (63.16) |  |
| Often | 833 (24.29) | 39 (24.07) |  |  | 334 (35.23) | 22 (28.95) |  |
| Everyday | 209 (6.10) | 10 (6.17) |  |  | 80 (8.44) | 5 (6.58) |  |
| Outdoor activities frequency (times per day), n (%) | | | <0.001 |  |  |  | 0.645 |
| ＜1 | 195 (5.69) | 24 (14.81) |  |  | 212 (22.36) | 15 (19.74) |  |
| 1-3 | 3083 (89.91) | 131 (80.86) |  |  | 695 (73.31) | 59 (77.63) |  |
| >3 | 151 (4.40) | 7 (4.32) |  |  | 41 (4.32) | 2 (2.63) |  |
| Outdoor activities time (min each time), n (%) | | | 0.828 |  |  |  | 0.152 |
| ＜15 | 284 (8.28) | 13 (8.02) |  |  | 47 (4.96) | 7 (9.21) |  |
| 15-30 | 1595 (46.52) | 71 (43.83) |  |  | 411 (43.35) | 33 (43.42) |  |
| 30-60 | 1138 (33.19) | 55 (33.95) |  |  | 344 (36.29) | 30 (39.47) |  |
| ≥60 | 412 (12.02) | 23 (14.20) |  |  | 146 (15.40) | 6 (7.89) |  |
| Sleep duration (hours per day), n (%) | | | <0.001 |  |  |  | 0.001 |
| ＜6 | 8 (0.23) | 6 (3.70) |  |  | 23 (2.43) | 5 (6.58) |  |
| 6-8 | 792 (23.10) | 32 (19.75) |  |  | 361 (38.08) | 28 (36.84) |  |
| 8-10 | 2349 (68.50) | 100 (61.73) |  |  | 559 (58.97) | 39 (51.32) |  |
| ≥10 | 280 (8.17) | 24 (14.81) |  |  | 5 (0.53) | 4 (5.26) |  |
| Bedtime, n (%) | | | 0.702 |  |  |  | 0.036 |
| Before 8 p.m. | 58 (1.69) | 3 (1.85) |  |  | 2 (0.21) | 2 (2.63) |  |
| 8-10 p.m. | 2877 (83.90) | 139 (85.80) |  |  | 818 (86.29) | 63 (82.89) |  |
| 10-12 p.m. | 484 (14.11) | 20 (12.35) |  |  | 128 (13.50) | 11 (14.47) |  |
| After 12 p.m. | 10 (0.29) | 0 (0.00) |  |  | 2 (0.21) | 2 (2.63) |  |
| Sleep quality, n (%) | | | 0.046 |  |  |  | 0.370 |
| Good | 2369 (69.09) | 99 (61.11) |  |  | 773 (81.54) | 65 (85.53) |  |
| General | 464 (13.53) | 23 (14.20) |  |  | 139 (14.66) | 7 (9.21) |  |
| Poor | 596 (17.38) | 40 (24.69) |  |  | 36 (3.80) | 4 (5.26) |  |
| Passive smoking (yes), n (%) | | | 0.763 |  |  |  | 0.394 |
| 1 | 233 (6.79) | 12 (7.41) |  |  | 63 (6.65) | 7 (9.21) |  |
| 2 | 3196 (93.21) | 150 (92.59) |  |  | 885 (93.35) | 69 (90.79) |  |
| Study time (hours per day), n (%) | | | 0.080 |  |  |  | 0.522 |
| ＜8 | 2254 (65.73) | 116 (71.60) |  |  | 302 (31.86) | 25 (32.89) |  |
| 8-10 | 991 (28.90) | 44 (27.16) |  |  | 491 (51.79) | 42 (55.26) |  |
| 10-12 | 163 (4.75) | 1 (0.62) |  |  | 132 (13.92) | 9 (11.84) |  |
| ≥12 | 21 (0.61) | 1 (0.62) |  |  | 23 (2.43) | 0 (0.00) |  |
| Extracurricular class (days per week), n (%) | | | 0.612 |  |  |  | 0.009 |
| Less one day | 2692 (78.51) | 125 (77.16) |  |  | 571 (60.23) | 59 (77.63) |  |
| One day | 691 (20.15) | 36 (22.22) |  |  | 364 (38.40) | 17 (22.37) |  |
| Two days | 46 (1.34) | 1 (0.62) |  |  | 13 (1.37) | 0 (0.00) |  |
| Digital products usage time (hours per day), n (%) | | | 0.533 |  |  |  | 0.488 |
| ＜2 | 1075 (89.88) | 70 (93.33) |  |  | 561 (76.64) | 65 (85.53) |  |
| 2-4 | 106 (8.86) | 4 (5.33) |  |  | 157 (21.45) | 11 (14.47) |  |
| 4-6 | 13 (1.09) | 1 (1.33) |  |  | 12 (1.64) | 0 (0.00) |  |
| ≥6 | 2 (0.17) | 0 (0.00) |  |  | 2 (0.27) | 0 (0.00) |  |
