# Supplementary material for: Capillary Glycated Hemoglobin A1c Percentiles and the Risk Factors Associated with Abnormal HbA1c among Chinese Children Aged 3–12 Years
Source: Pediatr Diabetes. 2024 Jul 29;2024:8333590. doi: 10.1155/2024/8333590 (PMC12017142; doi:10.1155/2024/8333590)
Supplement: Supplementary 5 — Figure S1: influencing factors of the HbA1c levels over the 95th percentile value of two subgroups (3–9 years old and 10–12 years old children) in the logistic regression model. [file 8333590.f5.docx]

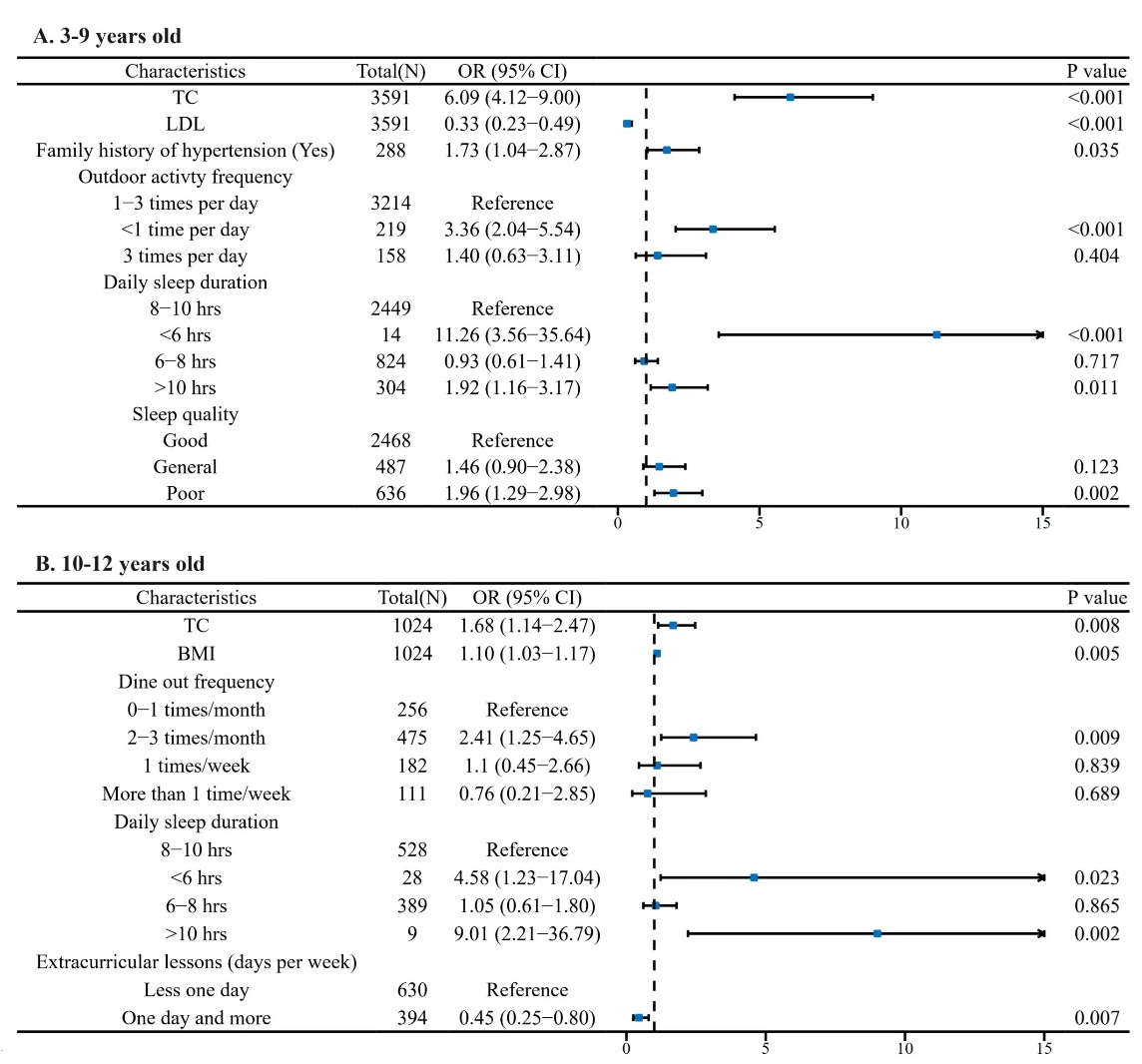


Figure S1: Influencing factors of the HbA1c levels over the 95th percentile value of two subgroups (3-9 years old and 10-12 years old children) in logistic regression model.
